# Nose-to-brain translocation of inhaled ultrafine elongated particles: facts and mysteries

**DOI:** 10.3389/ftox.2025.1655149

**Published:** 2025-11-20

**Authors:** U. M. Graham, J. M. Pinto, J. Weuve, A. K. Dozier, R. Rogers, S. Nag, J. Schneider, J. D. Kaufman, D. A. Bennett, G. Oberdörster

**Affiliations:** 1 BioInnovations Institute, Natick, MA, United States; 2 Medical Center, University of Chicago, Chicago, IL, United States; 3 Department of Epidemiology, Boston University, Boston, MA, United States; 4 Rush Alzheimer’s Disease Center, Chicago, IL, United States; 5 Medical Center, Rush University, Chicago, IL, United States; 6 Department of Environmental and Occupational Health Sciences, University of Washington, Seattle, WA, United States; 7 Department of Environmental Medicine, University of Rochester, Rochester, NY, United States

**Keywords:** nanofibers, axons, electron microscopy, inflammation, neurodegeneration, corpora amylacea

## Abstract

In this study, we report that inhaled nanosized elongated mineral particles (EMPs) reach the human central nervous system (CNS) via two neuronal pathways, cranial nerve I (olfactorius) and cranial nerve V (trigeminus), from deposits on the nasal mucosa. High-resolution analytical imaging of autopsied brain tissues from eleven members of a Religious Orders Study (ROS) cohort (Rush Alzheimer’s Disease Center) indicated that EMPs translocate from their nasal deposits to the brain either by the olfactory pathway (presence in the olfactory bulb (OB), olfactory tract, and amygdala) or by the trigeminal pathway (presence in the cerebellum). Sub-nanometer imaging and immunohistochemical (IHC) labeling were used to detect corpora amylacea (CA), abundant numbers of endogenous ferritin nanoparticles, and myelin damage as indicators of inflammation or oxidative stress. The majority of EMPs in the OB were identified as inorganic crystalline and amorphous SiO_2_ fibers. Amphibole-like fibers (Mg/Si/Fe) were present (length from 25 up to 200 nm), along with lengthened nanoplastics and metallic or carbonaceous fibers. Extensive and consistent demyelination, phosphorylation, wall thickening, and CA bodies (size ranging from 10 nm to ∼10 μm) are present in all studied brain tissues. EMPs are frequently observed inside and outside of CA bodies that occur in close proximity to neurons with myelin damage. The majority of EMPs show shedding of nanosized fiber fragments and ions from their long fiber surfaces and the formation of carbon-rich coronas (physiochemical alterations: bioprocessing). Similar to spherical nanoparticles, EMPs show a tendency to bioprocess, which involves interacting with microglia, astrocytes, and CA. In conclusion, we note that although the presence of ambient EMPs in the OB, amygdala, and cerebellum of human brains is consistent with neuronal translocation from nasal deposits of inhaled EMPs to the human CNS, it remains important to further investigate the potential contribution of nano-EMPs entering from the blood compartment by crossing the blood–brain barrier (BBB) and other potential routes to the CNS.

## Introduction and overview of neuronal brain circuitry

**FIGURE 1:**
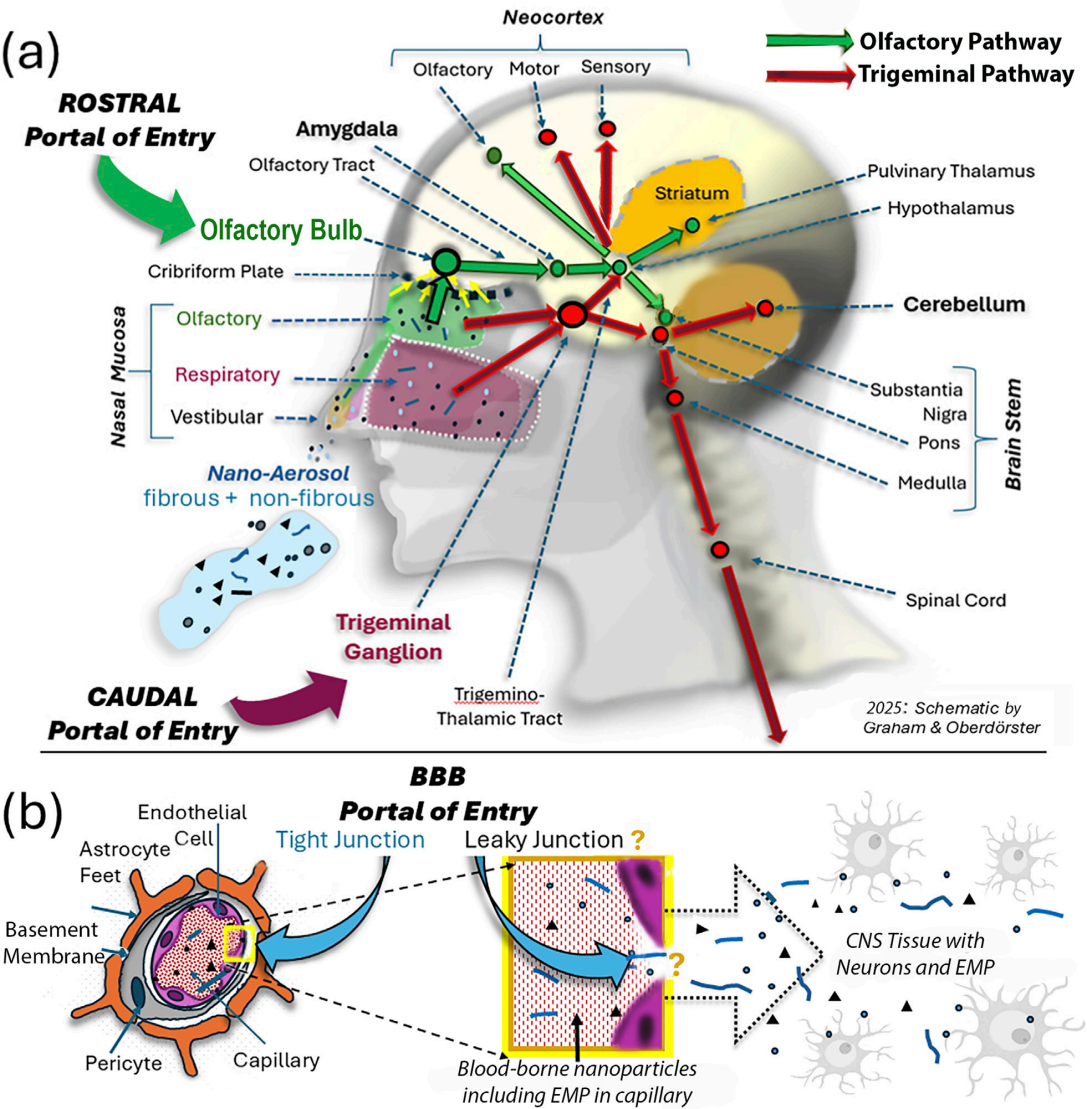
Schematic illustration of three portals of inhaled ambient nanoparticles, including EMPs, into the CNS. **(a)** Two extracerebral neuronal pathways of confirmed nose-to-CNS translocation for inhaled nanosized particles. From deposits on the olfactory nasal mucosa, these particles are translocated across the cribriform plate to the olfactory bulb via anterograde transport from the olfactory cells embedded in the nasal mucosa. Subsequent axonal transport from the OB along the olfactory tract to the amygdala and then continues to the hypothalamic region and the olfactory cortex (Chen et al., 2023). The trigeminal pathway originates both in the nasal respiratory mucosa and the nasal olfactory mucosa and then distributes to the caudal part of the brain, including the cerebellum beyond the trigeminal ganglion. **(b)** In addition to the two neuronal pathways illustrated in **(a)**, hypotheses have been proposed about additional entry routes to the CNS involving access of blood-borne nanosized particles that enter the blood compartment from deposits in the lower respiratory tract during air pollution episodes. The brain, through its micro-circulation, is very effectively protected by tight junctions between capillary endothelial cells (Engelhardt and Sorokin, 2009); however, with increasing age and under inflammatory conditions, brain endothelial tight junctions become increasingly leaky, so blood-borne nanosized particles, including EMPs, gain access to neurons in regions throughout the brain. Thus, depending on the degree of neurodegenerative diseases, the exogenous nano-EMPs observed in the caudal areas of the brain, indicated by the red colored arrows in **(a)**, have an entry path into the neuronal compartment that cannot be identified.

Fibrous and non-fibrous airborne particles have been the focus of numerous publications due to their effects in the respiratory tract following inhalation. These effects include devastating pulmonary fibrogenic and carcinogenic effects of inhaled asbestos, the toxic effects of man-made vitreous fibers (MMVFs), and, more recently, inflammation due to elongated mineral particles (EMPs). These studies are still ongoing, with the main emphasis placed on effects and underlying mechanisms in the respiratory system. Inhaled airborne particles have been found to reach the central nervous system (CNS) in both animals and humans (Elder et al., 2006; Maher et al., 2016). Only ultrafine particles in the range of ∼1–200 nm in ambient air have been found to translocate to the CNS. These were identified as “exogenous” particles in different regions of the brain, with the olfactory bulb (OB) as one portal of entry for a functional neuronal pathway, distributing these particles from deposits in the nose to the CNS (Maher et al., 2016; Teleanu et al., 2018; Teleanu et al., 2019; Faherty et al., 2025; You et al., 2022). A second neuronal “nose-to-brain” pathway exists via the trigeminal nerve, as shown in Figure 1 (Lochhead and Thorne, 2012; Saraiva et al., 2016). Given the frequent presence of fibrous-type particles in ambient air and knowing the inflammatory potential of inhaled elongated particles—e.g., asbestos in the respiratory tract—questions arise as to whether they can access the CNS by the same neuronal translocation route that is operational for nanosized spherical particles, and what their interaction is with target cells in the portals of entry, the olfactory bulb and the trigeminal ganglion, and in other subsequently deeper brain structures. Both ambient and occupational exposures may involve exogenous nano-EMPs that could enter the CNS. The current work is a proof-of-concept for EMPs’ neuronal translocation, providing the first evidence on this subject. The use of morphometric or volumetric analysis in brain tissue, optimistically, will be the subject of AI measurements.

The World Health Organization (WHO, 1985) has defined particles as fibers with a length of >5 μm, a diameter of <3 μm, and an aspect ratio of >3 : 1. This definition, which is also typically used for EMPs, which are designated as one-dimensional (1D) structures, does not apply to inhaled ultrafine fibers that translocate from deposits in the nose to the central nervous system via neuronal axon pathways, given a neuronal limit for axon diameter of ∼200 nm (Costa et al., 2018). Likewise, adverse responses in the respiratory tract have toxicologically been correlated with the “3Ds” (Oberdörster, 2000; Oberdörster et al., 2005), namely, dose (the number or mass of fibers deposited in the distal lung over time); dimension (fiber length and diameter); and durability (the persistence of fibers in the lung due to mechanical clearance and bio-dissolution). In general, this “3D” paradigm can illustrate nano EMPs that enter the brain. However, as pointed out above, the dimension of EMPs that will translocate to the CNS must be in the nanoscale range, yet with respect to their toxicity (dose) and persistence (durability), no quantitative data exist on the potential effects of EMPs that translocate to the brain.

Entries by inhalation of nanosized particles—including viruses—into the brain have been reported for laboratory animals and humans (Cheng et al., 2023; Jedličková et al., 2025; Vanbrabant et al., 2024; Wang et al., 2025; Hussain et al., 2020), with applications including drug delivery and the evaluation of neurotoxicity from airborne pollutants. Brain tissue is physiologically protected from even the smallest foreign particulates by the highly selective blood–brain barrier (BBB); age- and inflammation-related leakiness of brain capillaries can occur, particularly in disease states (Delaney and Campbell, 2017). Thus, airborne nanosized particles access the brain via two portals of entry: one via the olfactory pathway and the other via the trigeminal pathway. These transport routes are named after the two nerves, labeled the first (“olfactorius”) and fifth (“trigeminus”) extracranial nerve, that transport the nanoparticles inside their axons; thus, nanoparticles readily circumvent the BBB when being transported axonally with the first and fifth extracranial nerves (Chen et al., 2024; Handa et al., 2022; Tremblay and Frasnelli, 2018; Thorne et al., 2004). Figure 1 shows an overview of the olfactory and trigeminal translocation pathways for nanosized fibrous and non-fibrous particles from the nose to the brain in humans. In general, the deposition efficiency of inhaled micron-sized and larger fibers in the respiratory tract, including the nose, depends on their aerodynamic diameter and their dimensional characteristics (length and diameter), and they align with the main flow, with occasional rotations (Högberg et al., 2010). However, this alignment does not occur with nano-sized EMPs, so on average, there is no difference in nasal deposition between ultrafine spherical and elongated particles, the important determinants being aerodynamic and thermodynamic diameters. The site of nasal deposition is of importance for determining the fate of the deposited particles. There are three nasal mucosal areas (Figure 1): the vestibular mucosa at the tip of the nose, where cilia move deposited particles to the outside; the respiratory mucosa, the largest fully ciliated area, which is densely innervated by trigeminal nerve endings with cilia moving nanoparticles toward the nasopharynx and oropharynx; and the olfactory mucosa, which contains olfactory bipolar sensory neurons, with retrograde dendrites connecting to the nasal cavity and anterograde axons connecting through the bony cribriform plate to the olfactory bulb at the base of the frontal brain (Marin et al., 2018). Trigeminal nerve endings originate from both the respiratory and olfactory nasal mucosa to join along the path to the trigeminal ganglion and then distribute to cerebellar and other brain tissues (Figure 1a).

### Inhalation and EMP translocation from the upper respiratory tract via sensory nerves

Active endocytotic and pinocytotic activity of both the olfactory and trigeminal nerve cells facilitates the uptake of macromolecules and solid nanoparticles (Chen et al., 2024; Selvaraj et al., 2017), followed by axonal translocation along the two different pathways. For the olfactory pathway, it starts with pinocytotic uptake by dendrites in the nasal lumen, followed by retrograde movement to the olfactory cell embedded in the nasal mucosa, facilitated by transport molecules that include dynein, kinesin, actin, and myosin (De Lorenzo, 1970; Engelhardt and Sorokin, 2009). Subsequently, anterograde axonal translocation from the nasal olfactory neurons to the olfactory bulb within the cranium occurs across the protective cribriform plate (Figure 1a). This long plate plays another key role by providing open connections to the subarachnoid space and nasal lymphatic vessels around the olfactory nerves. These submucosal lymphatics in the nose connect to the main lymphatic vessels (Spera et al., 2023) and provide a direct link between the cerebrospinal fluid (CSF) and the lymphatic system in the nose. It constitutes a vital outflow pathway for clearing cellular and particulate debris in the CSF from the brain and directing it through the cervical lymph nodes into the bloodstream. Although the finding from this open link in a mouse study requires confirmation of its presence in the human cribriform bone, given the anatomical similarities among mammalian species, it may well be a convincing mechanism for particle-associated CSF clearance from the brain.

When comparing the “nose to brain” neuronal olfactory and trigeminal pathways, it becomes obvious that both together cover most brain regions (Figure 1). The two distinct pathways, illustrated as green and red arrows as lines with distinct stations (e.g., amygdala, pons, and cerebellum), indicate routes where the disposition of EMPs occurs depending on transport efficiency. The olfactory pathway (rostral portal of entry) starts at the bulb, continues as the olfactory tract to the amygdala—a center for emotional reactions, memory formation, and decision-making—and branches out into the thalamus for sensory processing (except smell) and to different structures of the neocortex. The trigeminal pathway (caudal portal of entry) starts at the trigeminal ganglion, where the ophthalmic, maxillary, and mandibular nerves join (Hunter and Dey, 1998), continues as the trigemino-thalamic tract to the pons and medulla regions of the brain stem, and connects to the cerebellum and the spinal cord (Figure 1a). Both pathways connect the substantia nigra and striatal structures, with inputs from the olfactory and the trigeminal branches, involving motor and sensory functions of the face and head. These branches are bilateral and additionally provide input to the midbrain and spinal cord (Figure 1). Importantly, the cerebellum is a part of the trigeminal pathway via the pons and substantia nigra. To the best of our knowledge, no prior publications have reported on neuronally translocated nanosized elongated particles. Although these descriptions of the two neuronal pathways are based on the neuronal communications in the CNS, we have only confirmed the presence of nanosized particles in the human CNS for specific stations along these sensory pathways. These include, for the olfactory pathway: OB, olfactory tract, amygdala; and for the trigeminal pathway, the cerebellum. In rodents (rats and mice), nasally administered nanoparticles were identified post-exposure in both the OB and trigeminal ganglion (Lochhead and Thorne, 2012; Hunter and Dey, 1998).

Our present high-resolution scanning transmission electron microscopy (HRSTEM) study documents the presence of elongated nanosized particles in eleven autopsied human brain tissues from the Religious Orders Study subjects (ROS cohort) (Bennett et al., 2012; Bennett et al., 2018; Grodstein et al., 2023). ROS is a longitudinal clinical-pathology investigation of Alzheimer’s and related dementia. Begun in 1994, its >1,500 participants are older nuns, priests, and brothers. At enrollment, they were living at more than 40 communal residences across the United States and were free of known dementia. These communal residences serve as study sites, offering an efficient means for participants to interact with study staff. ROS participants undergo annual standardized uniform evaluations for dementia and, upon death, undergo a brain autopsy (Bennett et al., 2012). The Institutional Review Board of Rush University Medical Center approved ROS. All participants signed informed and repository consents and the Anatomical Gift Act for brain donation.

### Study goals

Leveraging specimens from 11 ROS decedents, we detected EMPs in different brain regions and interpreted their location in the context of the two nose-to-brain translocation pathways depicted in Figure 1. Specific primary study objectives were to (1) identify any EMPs that are associated with the two pathways, the olfactory bulb/tract, amygdala, and cerebellum; (2) determine chemical composition and physical characteristics of EMPs to characterize their crystalline or amorphous and ambient air origins; (3) observe any interaction and bioprocessing (i.e., physiochemical alteration including dissolution and ion sheading from particle surfaces) of EMPs in different brain tissue regions with elemental mapping of the area; and (4) detect inflammatory or oxidative stress inducing signs such as the incidence of ferritin nanoparticles, destruction of myelin sheath, and incorporation of EMPs into corpora amylacea (CA) bodies, which are glycoprotein-based depositions that accumulate in aging and neurodegenerative diseases in the CNS. Thus, the study centers on analytical high-resolution imaging to probe the location (olfactory bulb, amygdala, and cerebellum), morphology (crystalline EMPs vs. amorphous fibers), chemistry, and redox activities of detected EMPs. This includes elemental mapping of the brain tissue regions surrounding translocated EMPs to investigate the physicochemical interaction, breakdown, or dissolution (bioprocessing) of the particles within the CNS tissues. It also includes monitoring endogenous ferritin nanoparticle accumulations that are linked to an oxidative response mechanism caused by invading exogenous nanoparticles (Graham et al., 2020). The HRSTEM studies were coordinated with immunohistochemistry (IHC) analyses of the olfactory bulb, amygdala, and cerebellar paraffin sections labeled with antibodies to image the presence of CA, neurofilament, and myelin. We documented the results obtained using the confocal microscope (Zeiss LSM 780) and recorded multiple labeled cellular components of the brain tissues to identify any EMPs. The goal was to find any spatial relationship between corpora amylacea, which are known to collect brain waste (Auge et al., 2018), and the presence of EMPs. The secondary goal of this study was to evaluate the results of the HRSTEM and IHC analyses tabularized together with available neuropathological findings to assess correlations and obtain preliminary data for designing a follow-up study. Since this study includes assessing a causal association between nano EMP/nano metals and risk of neurotoxic CNS effects, we performed HRSTEM and IHC analyses in a blinded design without prior knowledge of the individual subjects’ ages, genetics, and clinical neuropath diagnoses.

### Human brain tissue selection

As part of an exploratory study, for each of the 13 study sites set in the Chicago metropolitan area (sites listed by Bennett et al., 2012), we predicted annual ambient concentrations, from 1999 to 2013 (Keller et al., 2015), of three air pollutants: fine particulate matter air pollution (particulate matter <2.5 μm in aerodynamic diameter; PM_2.5_), nitrogen dioxide (NO_2_), and other NOx constituents. This particular model only addresses PM_2.5_, and no shape factors were involved in the PM_2.5_ NO_2_ study. There are no current PM_2.5_ models published that distinguish between particle shapes, including 1D structures. We selected eleven ROS decedents (Table 1) who had enrolled at Chicago-area sites representing a range of air pollutant concentrations. None had a history of smoking.

**TABLE 1:** Findings in the OB, amygdala (AM), and cerebellum (CER): Religious Orders Study ‘ROS.’

Sample	Pathological disease	Subject	APOE**	Braak	GPATH****	Corpora amylacea (CA)			Metals for all brain regions	Presence of EMPs			CA	Ferritin
Code*		age (yr)		score***		OB	AM	CER		OB	AM	CER		Next to EMP
#1	Left temporal lobe; chronic infarct	82	E3E3	III	0.1046565	+	+	-	Si, Al, Fe, Ti, Zn, Mg, As	Y		N	> CA	>>Ferritin
#2	NPD	80	E3E3	III	0.0949239	+	+	-	>Si, Al, Zn, Pb, Fe, As	Y	Y	Y	> CA	>>Ferritin
#3	Alzheimer’s	87	E3E4	V	1.3037992	+	+	+	>>Al, >> Fe, >Ti, Ni, Mg, Cu, <Pb,<Co	Y		Y	> CA	>>>Ferritin
#4	Alzheimer’s	88	E3E4	V	1.7096493	+	+	-	>>>Fe, >>Si, >>Al, Zn, Ti, <Ni, <Pb	N	N	N	> CA	None
#5	Alzheimer’s	88	E3E3	V	1.0631206	+	+	-	>>>Si, >>>Fe, >>Al, Zn, <Ni, <<Zr	Y	Y	Y	>> CA	>>Ferritin
#6	Alzheimer’s Lewy body	86	E3E3	V	1.4798907	+	+	+	>>Fe, >Si, >W, >Al, Zn, Cr, <Ni, <Pb	Y	Y	Y	>>> CA	>>Ferritin
#7	Alzheimer’s	90	E3E4	V	1.5059586	+	+	+	>>>Si, >>Al, >>Fe, >Pb, Ti, <Ni, <Mn	Y	N	Y	>>> CA	>>Ferritin
#8	NPD	90	E3E3	II	0.7967963	+	+	-	>>Fe, >>Si, >Al, Ti	Y	N	Y	>>> CA	>>Ferritin
#9	Alzheimer’s Lewy body	82	E3E3	VI	2.3074502	+	+	+	>>>Fe, >>Si, >>Al, >Pb, W, Ti, Ni, Mn	Y	Y	Y	>> CA	>>>Ferritin
#10	NDP	86	E2E3	I	0.0106854	+	-	-	> Fe, Si, Al, Mg	Y	N	N	> CA	>Ferritin
#11	Alzheimer’s	91	E2E3	IV	0.3404089	+	-	-	>>Fe, >Si, Ti, Mg, Zn, <Al	Y	Y	Y	> CA	>>Ferritin

*Tissue samples: Rush Alzheimer’s Disease Center (NDP, no dementia pathology).

**APOE: alleles: APOE2, APOE3, and APOE4 influence the genotype, and increasing genetic risk factors: protective (E2E2 and E2E3); neutral (E2E4 and E3E3); and highest genetic risk (E3E4 and E4E4). Subjects were genotyped by polymorphic DNA technologies (www.polymorphicdna.com) (Yu et al. 2017).

***Braak score: classifies Alzheimer’s disease-related severity and distribution of neurofibrillary tangle pathology (range I-VI) (Bennett et al. 2004).

****GPATH: quantitative measure of overall Alzheimer’s disease pathology burden (Bennett et al. 2006).

Metals, identified with HRSTEM–EDS.

OB, olfactory bulb; AM, amygdala; Cer, cerebellum.

## Analytical methods

### Brain tissue fixation for analytical imaging

The 11 OB tissue sections were submitted to HRSTEM/IHC analyses without initially revealing the disease status of the 11 subjects (Table 1) until the HRSTEM/IHC data collection, interpretation, and acceptance were complete.

The study, following clean removal of the brain, stabilized all tissues in 4% paraformaldehyde in 0.1 M phosphate buffer and processed blocks of olfactory bulbs, amygdala, and cerebellum using standard techniques and paraffin fixation. The study collected sections (6 µm thickness) on glass slides and transported them to the University of Rochester EM Laboratory for ultra-sectioning (thickness ∼25 nm). Tissue was lifted off microscopy slides followed by post fixation in two changes of 2.5% glutaraldehyde in 0.1 M Millonig’s sodium phosphate buffer (overnight at 4 °C). All slices were washed three times in distilled water, dehydrated in a graded series of ethanol, impregnated with polypropylene/epon-araldite mixtures, and embedded in 100% epon-araldite epoxy inside molds, followed by polymerization at 60 °C. The hardened tissue molds were then micro-sectioned using a diamond blade (Boeckeler PT-XL ultramicrotome from Boeckeler Instruments, Inc., Tucson, AZ) into 50–70 nm thin slices and mounted onto copper formvar grids stabilized with a carbon support layer (Ted Pella Inc., Redding, CA).

### HRSTEM and dispersive spectroscopy

The HRSTEM analyses differentiated between neuronally transported ultrafine EMPs (exogenous nanoparticles) and endogenous ferritin nanoparticles (biomineralized iron oxide and phosphate) based on morphological structures. To avert contamination of all 11-microtome blade-sectioned, polymer-embedded tissues, an argon glove box was used. The TEM sections were treated with a burst of high-pressurized, ultra-clean air in order to lift any loose particulates from the tissue section with a pressurized blast (moisture-free, oil-free, propellant-free, and residue-filtered compressed air; Chemtronics, United States) prior to the sections being mounted on either 2D or 3D sample holders and inserted into the JEOL scope. At sub-nanometer scales, ensuring that HRSTEM/EDS analysis accurately reflects the composition of the EMPs embedded in the extracellular matrix, we minimized hydrocarbon contamination from the environment or vacuum chamber, which is known to polymerize under the electron beam and obscure analysis. All sample holders are routinely plasma cleaned, and we also employ a baking phase to remove surface hydrocarbons from the thin sections prior to analytical imaging in order to avoid carbon buildup.

Translocated nanoparticles are distinguished from the background tissue by exploiting their intrinsic larger electron scattering cross-sections over those of the background tissue (which is typically composed of lighter atoms, particularly carbon and nitrogen), resulting in more X-ray generation. Henceforth, imaging in scanning transmission electron microscopy (STEM) using a high-angle annular dark-field detector (HAADF) results in larger numbers of electrons being scattered in X-ray energy-dispersive spectroscopy (EDS) analyses due to the much larger electron scattering cross-sections of the embedded nanoparticles within the host tissues. The pixel densities in line scans or maps across nanoparticles and the surrounding CNS tissue result in a substantial signal drop-off at the interface between tissue regions and the metallic particle’s composition. We further confirmed observations through correlative imaging coupled with morphological analysis. Since cryo-methods and cryo-sectioning were not available for this study, all tissue areas with evident surface imperfections (i.e., roughness from polishing and diamond blade-induced fractures) were excluded.

HRSTEM analyses were coupled with energy dispersive spectroscopy (EDS) for both line scans and mapping, using a JEOL 2100F Field Emission HRSTEM operated at 200 kV. The scope was equipped with an analytic pole piece, and high-resolution images were obtained using a Gatan Ultrascan 4K CCD camera managed by DigitalMicrograph software (Gatan, Inc.) and utilizing DigiScan II, Tridiem Gatan Image Filter (GIF), and Gatan HAADF. HRSTEM images were obtained with an analytical probe (0.17 nm). The Oxford Aztec EDS system (Oxford Instruments, Oxfordshire, United Kingdom) was coupled with Aztec STEM software (Oxford Instruments) to collect and manage EDS STEM data processing. EDS maps of EMPs within inside tissues were collected, and elemental concentrations were recorded within ∼2 min timeframe to minimize interactions and heating effects caused by the focused electron beam. Notably, no tissue staining was applied during the brain tissue fixation to avoid any potential interference with tissue-hosted metals, including EMPs, during HRSTEM analysis.

### Immunohistochemistry labeling of the olfactory bulb, amygdala, and cerebellum

Multimodal imaging and analysis of brain tissue sections involved a combination of regional, cellular, and subcellular analysis using immunohistochemical (IHC) labeling together with a corresponding correlation of systematic high-resolution analytical imaging (HRSTEM) of nanoparticles within the selected brain tissue sections. This approach made it possible to determine the locations and identities (i.e., chemistries and morphological features) of the nanoparticles within a precise anatomical context. SEM/EDS analyses of the corresponding EMPs microscopically observed were then compared to the IHC-labeled tissue sections investigated using fluorescent-labeled sections. This provides a subcellular biological context while comparing the localities of EMPs within the tissue sections in order to exclude any IHC labeling artifact caused by chemical interactions with the mostly metallic EMPs.

Our IHC investigation (Zaqout et al., 2020; Sun et al., 2021; Willemsen et al., 2021; Muranyi et al., 2022) used the AX/AX R Confocal Microscope System from Nikon Instruments Inc. on ∼6 µm-thick paraformaldehyde-fixed, paraffin-embedded (FFPE) olfactory bulb and amygdala sections lifted off glass slides. Sections were first deparaffinized and treated using an antigen retrieval (#62706, Electron Microscopy Sciences) unmasking step in Tris/EDTA buffer, pH 9.0 (AB93684, Abcam). Sections were then permeabilized and blocked with 5% goat serum (ab7481, Abcam) and a 1:10 dilution of human Fc blocking solution (130-059-901, Miltenyi Biotec) in 1X TBS-T. The sections were then immunoreacted overnight at 4 °C in a humidified chamber using a 1:100 mixture of primary antibodies starting with human IgM (CA, I8260, Millipore Sigma), followed by anti-neurofilament/NF-L antibody (Neural filament, ab223343, Abcam), anti-myelin basic protein antibody [EPR6652] (Myelin, ab133620, Abcam), and anti-GFAP antibody [5C10] (Astrocyte, ab190288, Abcam). Primary antibodies were washed off with 1X TBS. The secondary antibody was then applied at room temperature for 1 h using a 1:100 cocktail mixture of the appropriately cross-adsorbed secondary antibodies with spectrally compatible fluorophores: Alexa Fluor 488 AffiniPure Goat Anti-Human IgM, Fc5μ fragment specific (109-545-129, Jackson ImmunoResearch), Goat anti-Mouse IgG (H + L) Cross-Adsorbed Secondary Antibody, Alexa Fluor 633 (A-21050, Invitrogen), and Goat anti-Rabbit IgG (H + L) Cross-Adsorbed Secondary Antibody, Alexa Fluor 633 (A-21070, Invitrogen). After washing off excess secondary antibodies, slides were then cover-slipped with an anti-fade aqueous mounting medium (H-1900, Vector) and imaged using a Zeiss LSM 780 Confocal Microscope equipped with a 63X/1.4 Plan-Apochromat DICII objective for recording multiple labeled cellular components and foreign body constituents, with emphasis on detecting EMPs.

## Results

Table 1 provides a list of the selected ROS samples, numbered consecutively from 1 to 11, and indicates related pathological diseases and also findings in the olfactory bulb, amygdala, and cerebellum, specified by subject age and their APOE genotyping, indicating the associated degree of AD risk and overall AD pathology and correlation with Braak scores (Bennett et al., 2004; 2006). Unstained sections of the OB, amygdala, and cerebellum were provided by RUSH University to prevent any contamination that would interfere with the HRSTEM data collection.

Summarized in Table 1 are also the presence (+) or absence (−) of CA in the three different brain regions and the presence of EMPs in these areas, along with the overall frequency of CA occurrence in the three brain tissues. Considering the order of appearance, a listing of the nanosized metal inclusions is also provided, as is a side-by-side comparison of whether ferritin, alongside EMPs, was observed in the three different brain regions. Collectively, these comparisons will help evaluate which nose-to-brain neuronal pathways, either the rostral or caudal portal of entries, were the likely translocation pathways if nose translocation was the only access. This knowledge will help better evaluate CNS transfer routes of exogenous EMPs (Figure 1).

An in-depth evaluation, including statistics of the combined findings in Table 1, could help reveal relationships between the degree of clinical pathology and the presence of nanosized EMPs and metals in all brain regions: For example, if a sufficient number of study subjects show no AD pathology and only the lowest Braak score, with no evidence of EMPs, ferritin, or CA bodies in the three investigated brain regions, this might be indicative of a causal relationship. Subject #10 in Table 1 represents one example of this case. Subject #8 also shows low AD pathology and a low Braak score, yet exhibits low Pb levels, with EMPs detected only in the OB tissue. In contrast, all other subjects show different degrees of AD pathology with varying amounts, or even no EMPs (subject #4), and EMPs together with various amounts of nanosized Fe and other metals. Caution needs to be exercised when drawing premature conclusions from a low-number dataset. Contrary to the conclusion of a causal relationship between the number of CA bodies in the OB, amygdala, and degree of AD (Bragg Score), subject #4 shows minor CA body formation in these brain sections despite having suffered from significantly advanced AD. Thus, at present, the results in Table 1, based on only 11 subjects, are insufficient to assess a causal association of nano-EMP in the CNS with AD. Still, the subjects with no or low AD pathologies may provide necessary control data with respect to a CNS-risk from inhaled nano-EMP and nano-metals. It is therefore still valuable to examine such association in an appropriately designed study with a sufficient number of cases including matching controls.

**FIGURE 2:**
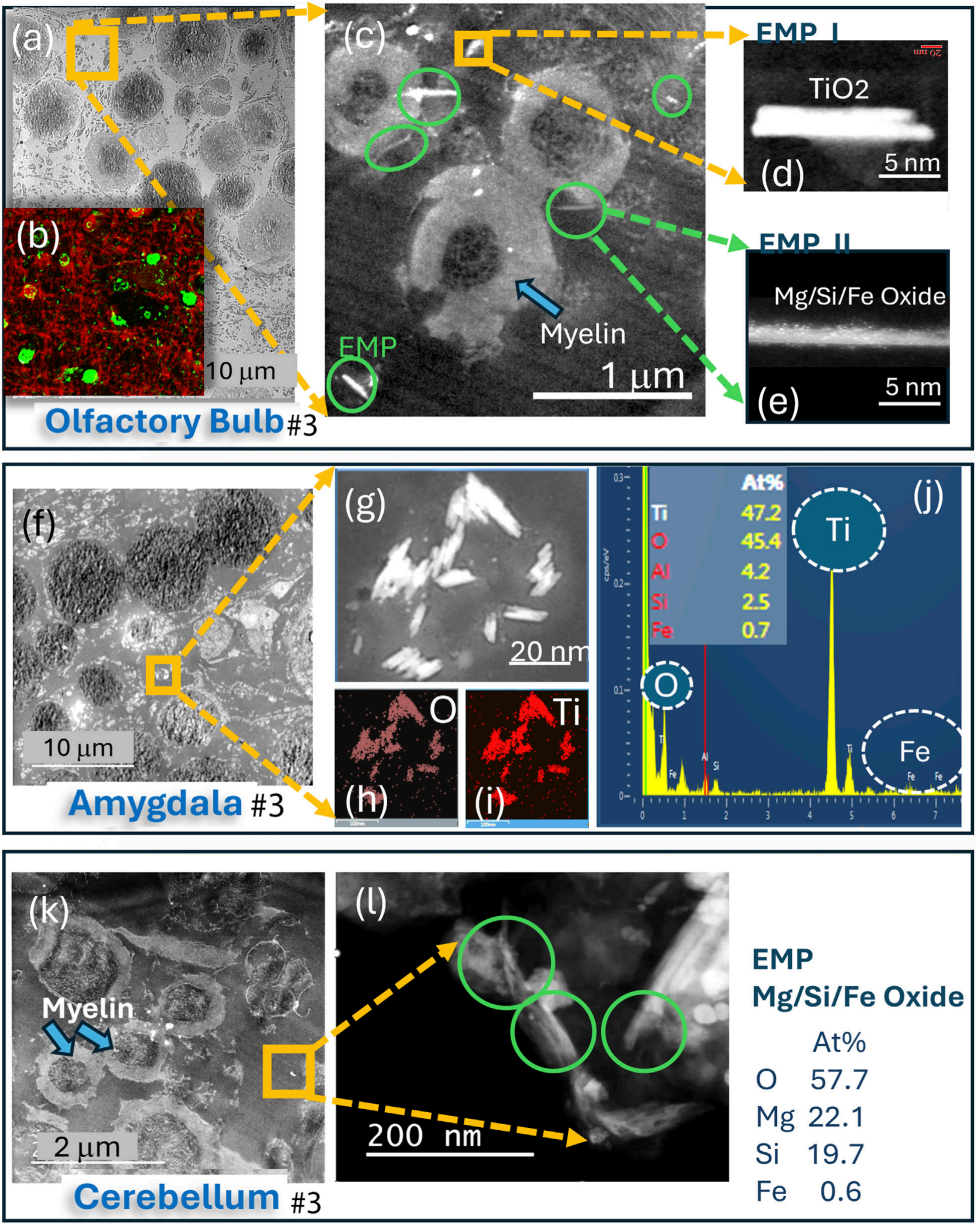
Analytical imaging of corpora amylacea (CA) and EMP inclusions in the olfactory bulb, amygdala, and cerebellum tissue regions of subject #3: **(a–l)** HRSTEM and EDS analyses and mapping results for all three CNS brain regions. HRSTEM analyses illustrate the presence of EMPs and corpora amylacea. **(a)** HRSTEM and **(b)** IHC illustration of corpora amylacea in the olfactory bulb; **(c)** HRSTEM of EMPs next to neurons with thickened and vacuolated myelin sheets; **(d)** magnified TiO_2_ EMP; **(e)** magnified amphibole-like EMP. **(f)** HRSTEM of the amygdala with corpora amylacea bodies and cluster of EMPs; **(g–j)** magnified EMP with EDS maps for O and Ti and EDS spectrum with elemental analysis for TiO_2_ cluster. **(k)** HRSTEM of the cerebellum with a myelinated area and no corpora amylacea bodies but EMP clusters between neurons that have damaged myelin sheets; **(l)** magnified view of EMP cluster marked in the yellow square in **(k)** and corresponding EDS analysis for EMP composition.

The presence of EMPs, as identified by HRSTEM analyses, is summarized in Table 1 to show their occurrence and distribution across the different brain regions. In general, the majority of EMPs were found in the olfactory bulb and included EMPs of inorganic origin, such as SiO_2_ (both crystalline and amorphous) and amphibolic fibers with Si–Mg composition and minor Fe components, along with carbon-rich EMPs that include carbon filaments and plastic nanofibers. Specific examples are shown for EMPs analyzed inside subject #3’s olfactory bulb (Figures 2a–e), amygdala (Figures 2f–j), and cerebellum (Figures 2k,l), which compare for the first time not only the presence but also the composition and structure of the EMPs in the cerebrum and cerebellum, confirming that not only spherical but also elongated nanosized particles reach both portals of entry sites and deeper brain regions, as verified in the same subject (in this case, subject #3; see Table 1). Furthermore, our HRSTEM investigation shows corpora amylacea in the olfactory bulb (Figure 2a) and marks the locations where EMPs are found, while our corresponding IHC labeled tissues show the distribution of copious corpora amylacea (∼2–50 μm) throughout the olfactory bulb with broken-down myelin (Figure 2b). A higher magnification is shown in Figure 2c, which illustrates the presence of EMPs next to neurons showing demyelination, thickening, and major vacuolization, resulting in myelin debris formation. Two of the EMPs in Figure 2c are imaged in higher magnification in HRSTEM in Figure 2d and identified as TiO_2_ EMPs that are approximately 13 nm long and 2 nm wide and in Figure 2e as a Mg/Si/Fe-type EMP (composition similar to that of amphibole asbestos) that has a high aspect ratio (∼25) and is 3 nm wide and ∼75 nm long. An HRSTEM documentation for the presence of EMPs within the amygdala for the same subject # 3, who had TiO_2_ EMP present in the olfactory bulb (Figure 2d), revealed that copious TiO_2_ EMPs were present inside or juxtaposed to corpora amylacea (Figure 2f). The TiO_2_ EMPs are magnified further in Figure 2g, with corresponding elemental maps shown for O (Figure 2h) and Ti (Figure 2i), with particle sizes similar to those in the olfactory bulb tissue, having a length scale of ∼10–20 nm and a width of ∼ 3–5 nm. A corresponding EDS spectrum taken on a cluster of TiO_2_ EMPs is shown in Figure 2j and indicates that Ti (47.2 At%) and O (45.4 At%) are the dominant elemental components, and some minor concentrations of Al (4.2 At%), Si (2.5 At%), and Fe (0.7 At%) are present as part of the total tissue spot analysis over the EMP cluster. We observed several Mg–Si EMPs (similar composition to that of asbestos) with Mg (22.1 At%), Si (10.7 At%), Fe (0.6 At%), and O (57.7 At%) in the cerebellum tissue sample (Figures 2k,l). These are similar in size and aspect ratio to those observed in the olfactory bulb of the previously mentioned subject #3 (Table 1). The olfactory pathway lines up in close proximity with the OB, olfactory tract, and amygdala that were analyzed, and the fact that the Ti-rich fibers in OB and amygdala show exactly the same chemical composition provides evidence that they are derived from the same source and that this neuronal translocation pathway operates in humans.

**FIGURE 3:**
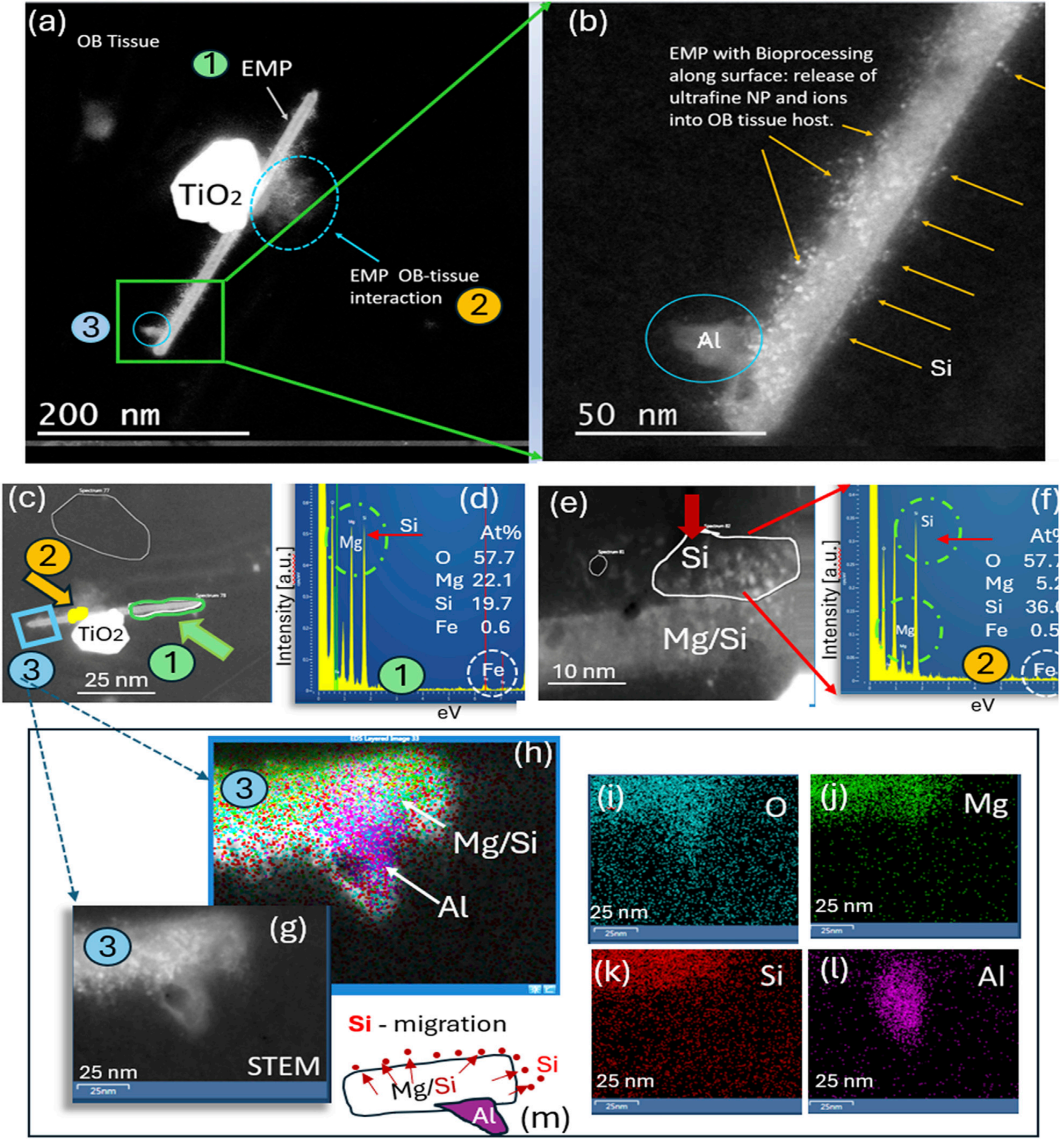
Analytical imaging of EMP inclusions in the olfactory bulb tissue of subject #3: **(a)** HRSTEM image of EMPs in olfactory bulb tissue attached to a TiO_2_ nanograin; **(b)** magnified view of EMPs with Si nanoparticles present on top of the fiber and protruding into the adjacent olfactory bulb tissue region and an Al grain attached onto the fiber. **(g–l)** HRSTEM and corresponding elemental maps for a region of the EMP shown in **(a–c),** illustrating that the EMP fiber consists of Mg/Si-rich region in the core, while Si is further enriched along the fiber surface; the Al nanograin is intergrown with the EMP fiber rather than attached to the surface, as illustrated in **(h)** and **(l)**. **(m)** Schematic illustration of the Si migration from the EMP bulk to the surface layer of the fiber and also into the surrounding tissue, thereby releasing Si into the olfactory bulb.

An HRSTEM analysis of an Mg–Si EMP (EDS analysis indicates composition similar to that of asbestos) undergoing tissue interactions in the olfactory bulb of ROS subject # 6 (Table 1) is shown in Figure 3. The results show that the fiber surface sheds ultrafine particles and potentially ions into the surrounding olfactory bulb tissue (Figures 3a,b). This interaction or bioprocessing along the EMP surface (a) reduces the EMP’s overall size, (b) alters the chemical composition of the EMP’s surface layer, and (c) alters the olfactory bulb tissue composition next to the EMP. Figure 3a depicts three different regions of interest of the EMP. A nanosized TiO_2_ particle is positioned on top of the fiber, and where the particle and fiber intersect, there is a noticeable debris formation and tissue interaction. The lower end of the EMP fiber is further magnified in Figure 3b, which shows that the surface has a substantial number of ultrafine nanoparticles and electron-dense regions, likely due to ion release from the EMP surface layer into the surrounding OB tissue. The particle debris along the fiber axis was analyzed to be Si nanoparticles ranging anywhere from 1 to 6 nm in size. A larger Al-rich fragment is attached to the EMP, and Figure 3c shows three regions along the EMP axis, where EDS analysis was conducted, while Figure 3d shows the chemical composition of the EMP to include Mg (22.1 At%), Si (19.7 At%), Fe (0.6 At%), and O (57.7 At%) (composition corresponds to amphibole asbestos). The EMP surface that had the greatest bioprocessing region is shown in the HRSTEM image in Figure 3e. Specifically, individual Si-nanoparticles populate the surface at the olfactory bulb tissue interface region. Some areas on the fiber surface appear porous or leached out, and a corresponding EDS spectrum in Figure 3f of the Si-rich surface region indicates that Mg is drastically reduced for Mg (5.2 At%) and enriched in Si (36.0 At%) with minor Fe (0.5 At%) present. The process of Si-migration to the surface of the Mg/Si/O EMP is shown in the HRSTEM and corresponding elemental maps in Figures 3g–l and schematically depicted in Figure 3m, where Si nanoparticles released from the EMP bulk migrate to the surface and thereby change the surface composition, which is in contact with the olfactory bulb tissue, a dynamic process that could alter the nanotoxicological fingerprint of the EMP and potential inflammatory responses.

**FIGURE 4:**
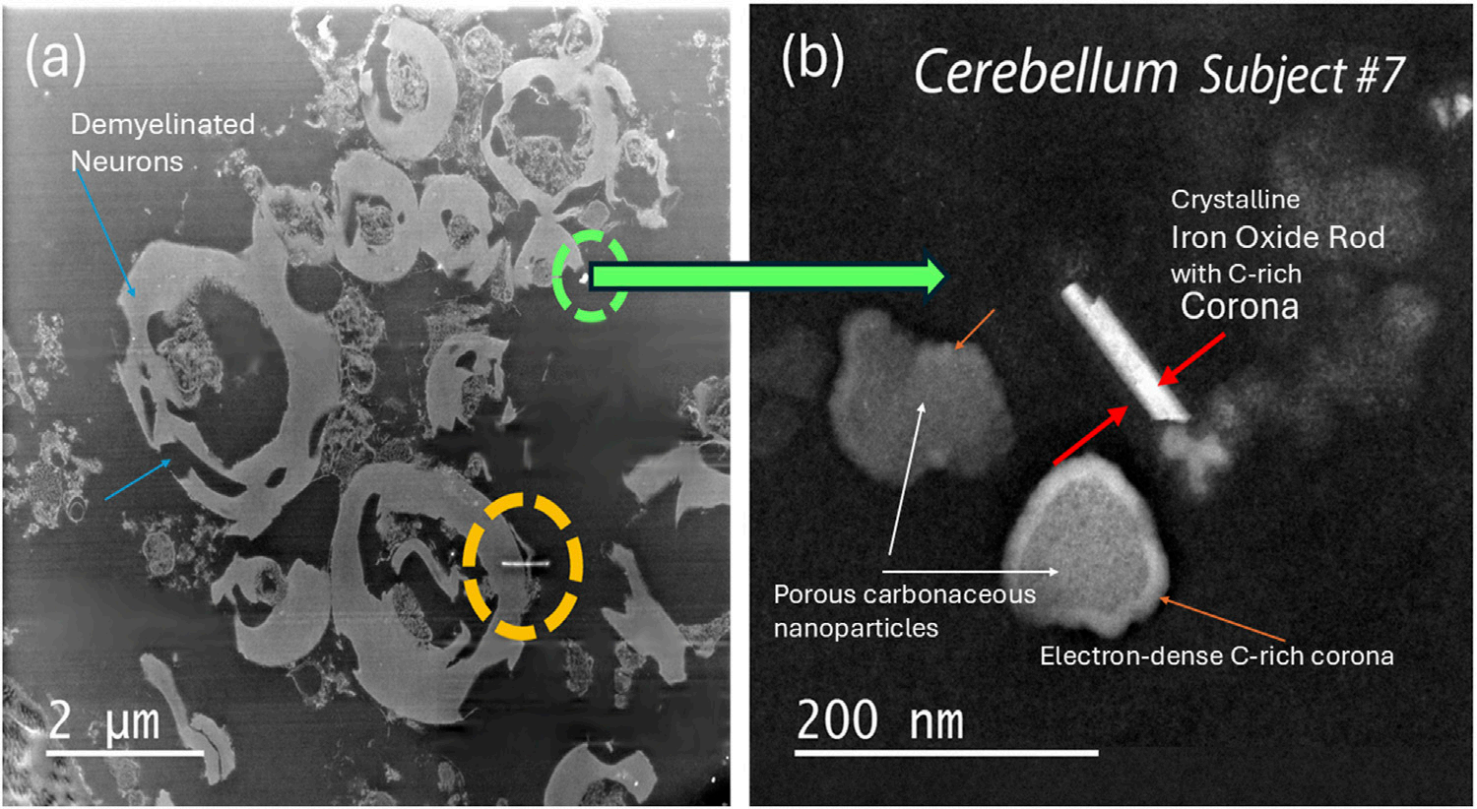
Analytical imaging of EMP inclusions in the cerebellum tissue of subject #7 **(a)** HRSTEM imaging of the cerebellum tissue region that includes multiple translocated EMPs between neurons that have undergone de-myelination. The green and yellow circles mark the area where EMPs can be observed even at the low magnification setting; **(b)** magnified view of an EMP marked with a green circle identified in **(a)** that is in close proximity to neurons with myelin damage. The EMP is magnified in **(b)** and corresponds to an inorganic exogenous iron-oxide crystalline rod with a ∼5 nm thin corona, indicating EMP–tissue interaction occurred along the surface of the EMP. Nearby carbonaceous particles also show a narrow ∼10–15 nm thin carbon corona.

Demyelination, including myelin swelling, significant vacuolization and myelin sheet debris, is demonstrated in the HRSTEM (Figure 4a) of cerebellum tissue derived from subject # 7 (Table 1). The image shows several nanoparticle inclusions comprised of spherical and angular nanoparticles, including EMPs, which are marked by green and yellow circles in 4a, as well as in the magnified HRSTEM (Figure 4b). The latter illustrates a proteinaceous/carbonaceous corona formed around the inorganic exogenous iron oxide crystalline nanorod (EMP) with a ∼5 nm thin carbon corona, indicating that EMP–tissue interaction occurred along the surface of the EMP. Nearby carbonaceous particles also show a narrow ∼10–15 nm thin but electron-dense carbon corona.

**FIGURE 5:**
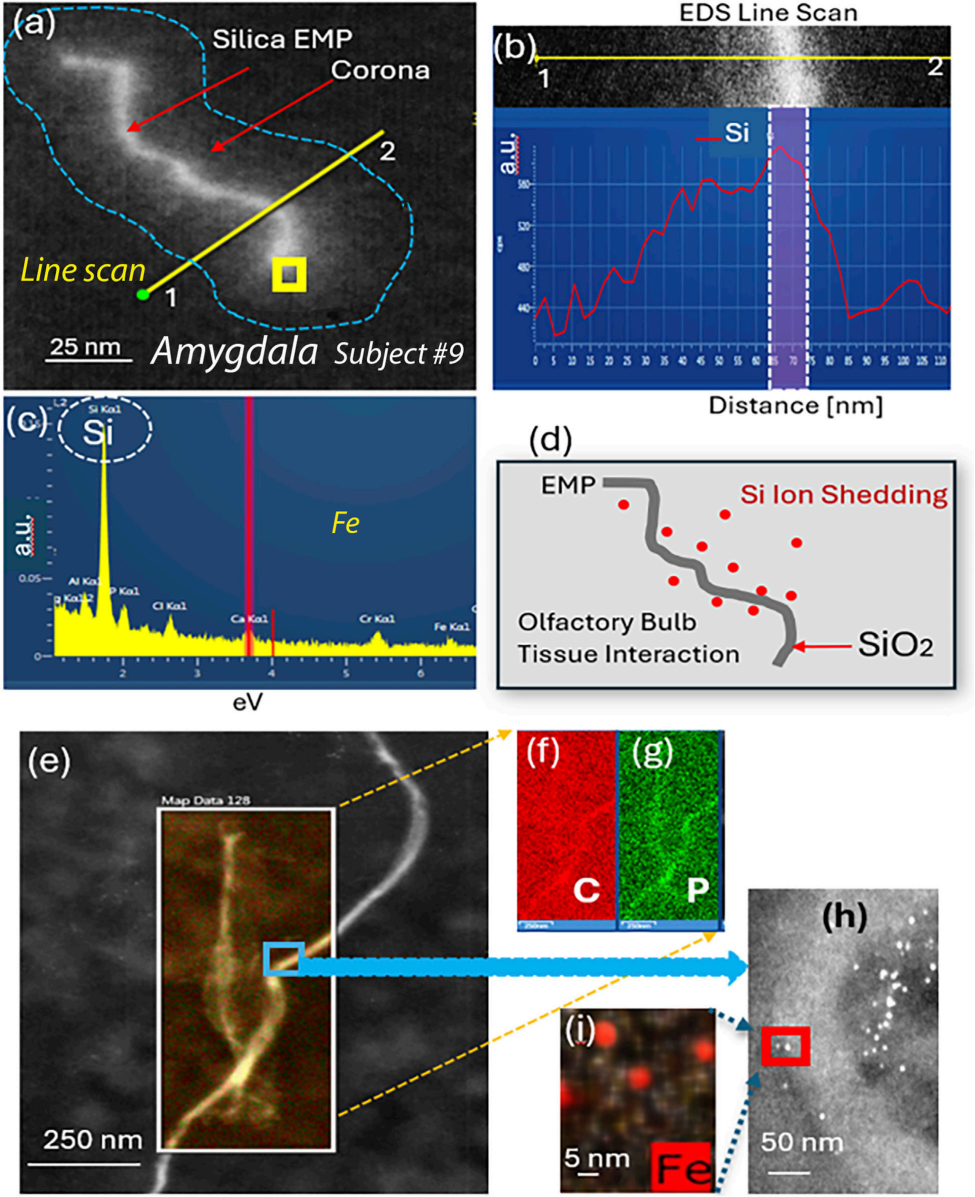
Analytical imaging of EMP inclusions in the amygdala tissue of subject #9: HRSTEM and EDS mapping of silica EMP **(a–d)** and carbon EMP **(e–i)**. **(a)** Silica EMP has a corona formation, and an EDS line scan (yellow line) is shown to traverse from outside of the corona, across the corona region and EMP toward the opposite side and back out of the corona region, which is illustrated in **(b),** showing the distance for the line scan with corresponding Si signal strength. The yellow square in **(a)** shows where EDS analysis shown in **(c)** was carried out. **(d)** Schematic showing the Si ion shedding process where Si is liberated off the SiO_2_ fiber surface and migrates into the surrounding olfactory bulb tissue interface region. **(e)** HRSTEM of carbon fibers in the olfactory bulb with corresponding EDS maps for C and P; **(h)** magnified view from blue square in **(a),** showing the presence of ferritin nanoparticles next to EMP; (i) EDS map for Fe for a select region in **(h)** showing ferritins.

**FIGURE 6:**
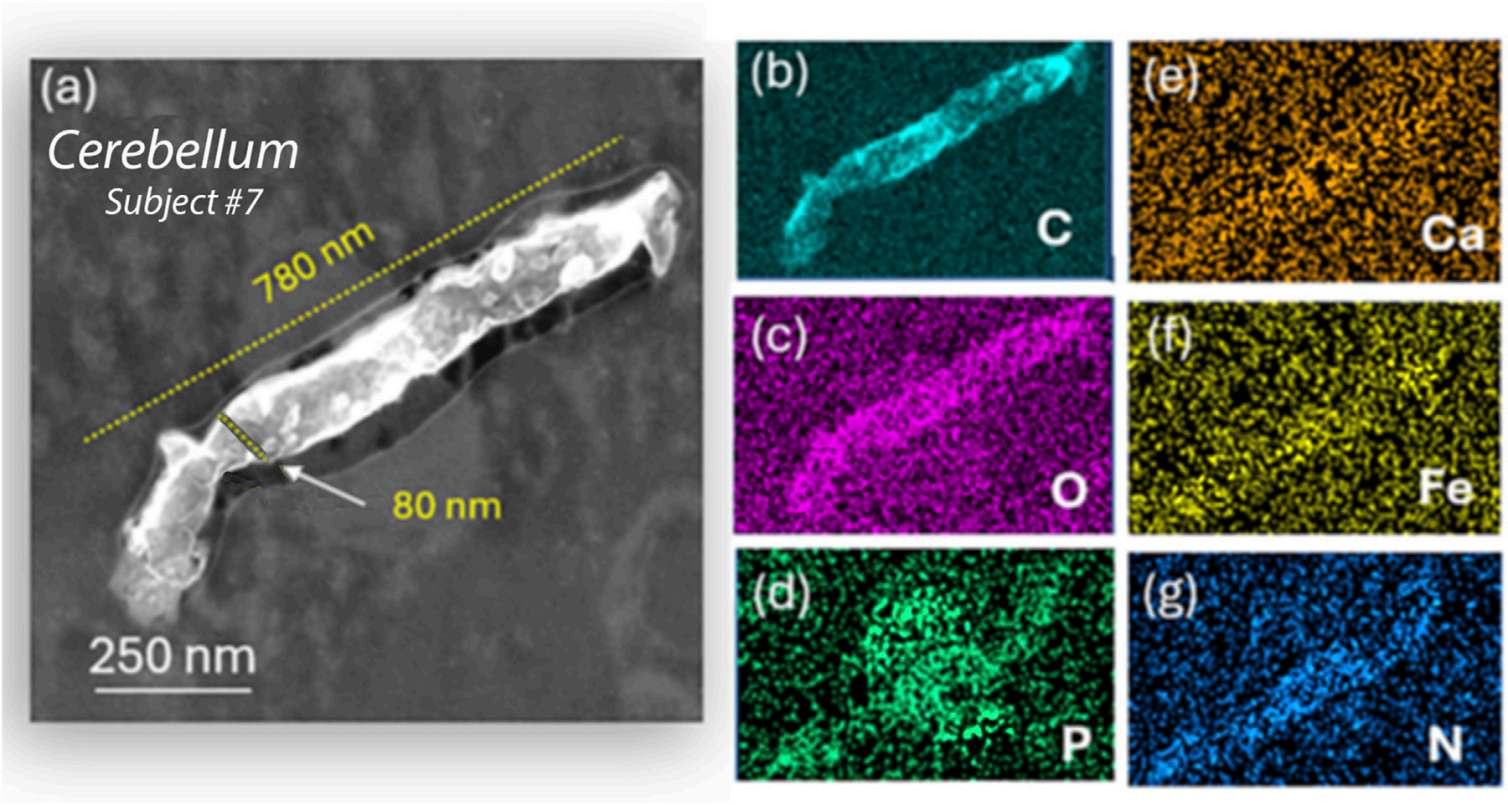
Analytical imaging of nano-plastic fiber inclusions in the cerebellum tissue of subject #7: **(a–g)** HRSTEM and corresponding EDS maps for carbon, calcium, oxygen, iron, phosphor, and nitrogen show a large carbon- and nitrogen-dense fiber inside the cerebellum tissue, which reacts with the 200 keV electron beam, creating a rough surface on the nanofiber. The EDS signal corresponds to that of a standard polyethylene fiber but shows phosphate infiltration extending from the cerebellum tissue into the nanofiber core.

EMP–tissue interactions are further proven with HRSTEM elemental line scan analysis in the amygdala of ROS subject #9, which is shown in Figure 5a for a wavy silica (SiO_2_) EMP fiber with amorphous structure. The SiO_2_ EMP is surrounded by a zone that is not a carbon corona but rather a Si-rich corona. The EDS line scan analysis is illustrated in Figure 5b, which documents the elemental composition of the EMP fiber along the axis and the corona regions on both sides of the EMP fiber. A Si-rich zone is shown to reach >50 nm distance from the fiber axis into the amygdala tissue. A single EDS analysis is carried out directly on the EMP, and the result in Figure 5c confirms that the EMP is a silica fiber. The Si-component within the amygdala tissue is ionic rather than nanoparticulate, indicating that ion shedding from the fiber axis occurred and diffused into the surrounding host tissue (amygdala), as schematically shown in Figure 5d. The HRSTEM and elemental maps of several carbon EMPs (Figures 5e–g), which were discovered in the olfactory bulb tissue of ROS subject # 11 (Table 1), showed a small tissue buildup of P immediate at the EMP–tissue interface, suggesting that the EMP was not a stealth inclusion but rather caused a tissue response. At higher magnification, the HRSTEM analysis and elemental map for Fe showed the presence of ferritin nanoparticles at the carbon EMP–tissue interface (Figures 5h,i), which is often observed when invader particles are associated with an oxidative stress response (Graham et al., 2020). The study also observed a very large, 780-nm-long and 80-nm-wide EMP particle in the cerebellum (subject # 7) (Figure 6), which is an integral part of the cerebellum tissue with bioprocessing effects and not postmortem contamination. The EMP is extracellularly present in the cerebellum. The corresponding HRSTEM analysis with EDS maps for C, O, P, Ca, Fe, and N shows that the EMP has a heterogeneous composition compared with the carbon-based EMP shown in Figure 5. The particle has a strong outer wall consisting of C, O, P, and N (Figures 6a–d,g), resembling those of hollow plastic nanofiber EMPs. There is a strong overgrowth of P, Ca, and Fe (Figures 6d–f), which suggests a particle–tissue interaction occurred in the living person, involving iron phosphate together with the accumulation of ferritin nanoparticles (biomineralized iron).

**FIGURE 7:**
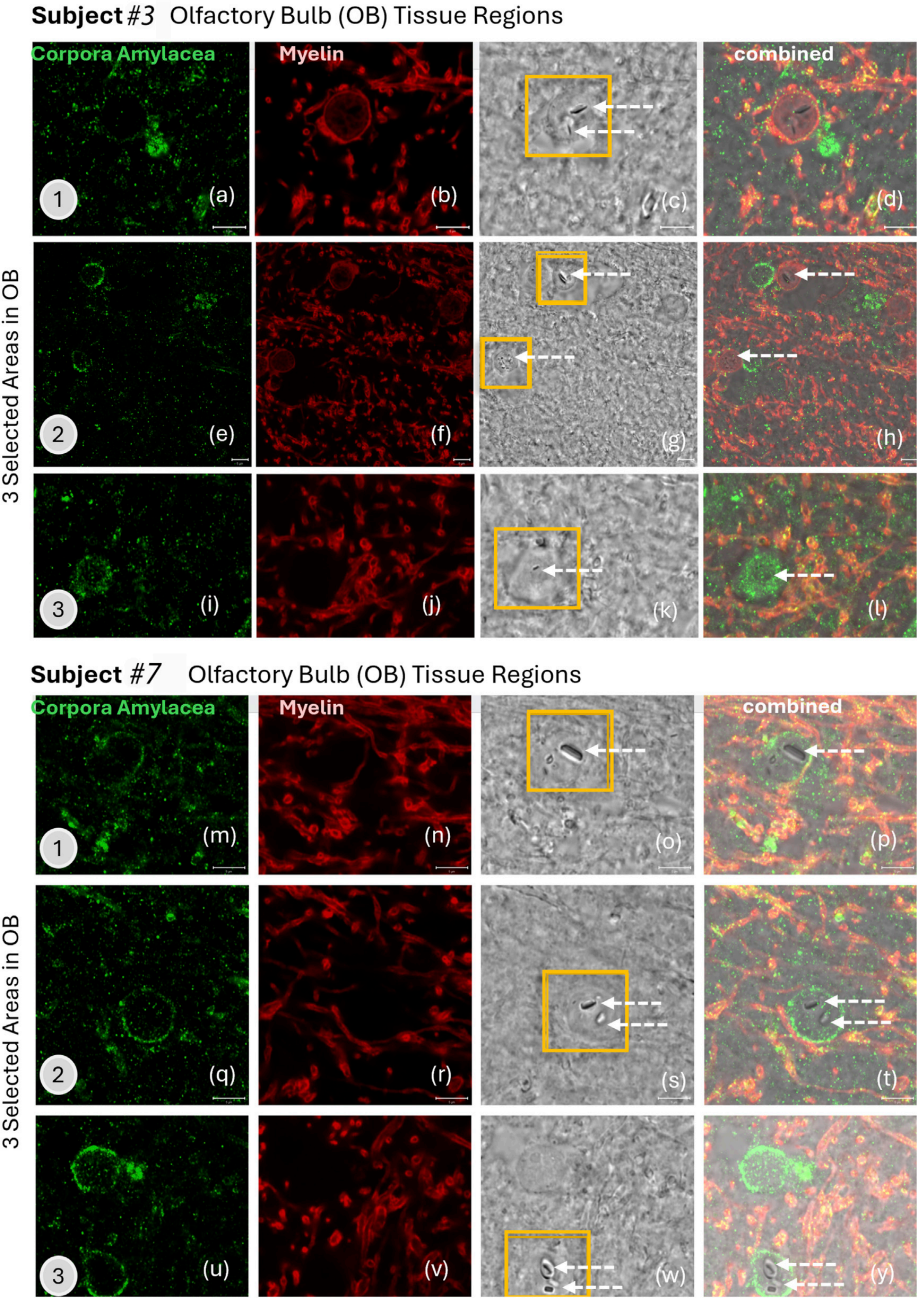
**(a–y)** IHC labeling of (green) corpora amylacea (human IgM, I8260 Millipore Sigma) and (red) myelin (anti-myelin basic protein antibody [EPR6652]; myelin, ab133620, Abcam) in the olfactory bulb tissue of two human subjects are illustrated with confocal microscopy images. Three selected areas are presented for subjects #3 and #7 (subjects listed in Table 1). White arrows show the locations of EMPs in the grey-scale images that are from the exact same tissue location as the stained images.

The biological response of individual EMPs in the olfactory bulb, amygdala, or cerebellum cannot be distinguished at this time since the tissues also contained copious non-fibrous metal nanoparticles. The interaction of nanoparticles with brain tissue depends strongly on the physicochemical properties of the particles—in the case of EMPs, their 1D morphology—and on fiber size (aspect ratio) and shape. The surface charge and composition will play significant roles in determining the tissue interactions, such as ion shedding from the particle surfaces into the host CNS tissue. All particles must be considered when investigating the inflammatory state of the different brain regions and the number of CA observed (Augé et al., 2018; Riba et al., 2021). To associate the presence of EMPs with CA-accumulation in different olfactory bulbs, we performed IHC analyses. These included two different ROS subjects (see Table 1 for subjects #3 and #7), with three distinct regions within the corresponding olfactory bulb tissues for each subject. The results are shown in Figures 7a–y. Importantly, the IHC data identify a biological response mechanism observed in the olfactory bulb of the human subjects, where copious CA formed spherical and semi-spherical polyglucosan containers (Auge et al., 2018; Riba et al., 2021) with a granular appearance. In the IHC-labeled confocal microscopy images, which were specifically stained to reveal the presence of CA (human IgM, I8260, Millipore Sigma) for the first subject (Table 1. Subject #3; Figures 7a,d,g) and the second subject (Table 1. Subject #7; Figures 7m,q,u), CA structures were identified. Ultrastructurally, these bodies are characterized by randomly arranged and interlocked polyglucosan fibers that are chemically and structurally distinct from neurofilament tangles (Auge et al., 2018). However, these ultrastructures cannot be resolved by confocal microscopy. Our study explicitly used the IHC labeling approach to localize and image the CA over larger tissue regions and show them alongside the myelinated neurons for subject #3 (Table 1; Figures 7a–l) and subject #7 (Table 1; Figures 7m–y). The corresponding gray-scale images are also included in Figure 7 and show the locations of EMPs in the tissues (white arrows). The results clearly show that multiple EMPs, as identified with white arrows in Figure 7, are spatially associated with CA. SEM/EDS analyses of the corresponding EMPs microscopically observed were then compared to the IHC-labeled tissue sections investigated using fluorescent-labeled sections. This provided a subcellular biological context while comparing the localities of EMPs within the tissue sections in order to exclude any IHC labeling artifact caused by chemical interactions with the metallic EMPs. Since these findings show, for the first time, that CA bodies incorporate EMPs in a similar fashion to what we observe for diverse spherical nanoparticles. The mechanism by which CAs incorporate EMPs is not known. However, it appears that this incorporation isolates the invading nanoparticles from exerting further potentially adverse interactions with sensitive target cells in the brain.

## Discussion

Our serendipitous discovery of a single high aspect ratio (∼25) amphibole-type nanofiber within the human brain showed that this nano-EMP had already undergone bioprocessing and physicochemical interactions with the OB tissue, rather than being deposited onto the sample surface post-fixation—an observation that supports neuronal transport of inhaled nano-EMPs. After this first encounter and with a more trained eye, several nano-EMPs, including inorganic metal oxide and plastic nanofibers, are successively detected in the human OB, amygdala and cerebellum, confirming the olfactory pathway for transport of inhaled “1D” nanostructures (nanofibers, nanorods, and nanocrystals: “nano-EMPs”). The OB transport route was previously shown to operate for inhaled non-fibrous particles, but only of nanosize (Maher et al., 2016; Graham et al., 2020). Much larger non-fibrous and fibrous plastic microparticles (several micrometers in diameter/length) were recently shown by Amato-Lourenco et al., 2024, and nanosized particles (Graham et al., 2020) suggest different transportation paths from the airborne state to OB deposition that should be addressed in future studies. Using micro-Fourier transform infrared spectroscopy, these particles were found to be present inside human OB tissues. This extraordinary difference in size between the micro-sized particles (Amato-Lourenco et al., 2024) and nanosized particles highlights distinct translocation mechanisms.

We used HRSTEM analytical imaging and EDS mapping to trace exogenous nano-EMPs ranging from ∼10 to 200 nm in length and 2–25 nm in width (Figures 2–5) and their subsequent distribution to different sections of the CNS in 11 human subjects (Table 1). To the best of our knowledge, the current study is the first demonstration of nanoscale 1D-structures, including TiO_2_, SiO_2_, Fe_2_O_3_, and Al_2_O_3_, and amphibole-type mixed metal oxides (Figure 2), along the carbon and plastic nanofibers (Figures 5, 6) in the OB, amygdala, and cerebellum. In particular, the amphibole nanofiber surface composition (O:Mg:Si:Fe; at% 57:5:36:0.5) was enriched in Si compared to the nanofiber’s bulk composition (O:Mg:Si:Fe; at% 56:22:19:0.5), and Si migrated further into the OB-tissue at the nanofiber–tissue interface (Figure 2). This raises questions regarding the potential adverse nanofiber effects throughout the CNS when inhaled. Nanoplastic fibers (Figures 5, 6) also showed tissue interactions and the onset of bioprocessing in the amygdala (Figure 5) and cerebellar region (Figure 6), with some porosity increase in the nanofiber structure and enrichment of phosphate at the tissue–nanofiber interface with some infiltration of phosphate into the fiber core (Figure 6). Many nanoplastic fibers were surrounded by endogenous ferritin particles—a hallmark response to the upregulation of iron at the tissue interface of the invader nanofiber, and ferritin buildup is an indicator of stress response and inflammation typically observed with non-fibrous nanoparticles (Graham et al., 2017; Moreira et al., 2020). The HRSTEM data enabled precise localization of nanofibers within human brain tissues and revealed their spatial associations with neurons and CA bodies—findings that, compared with digest-based analyses, show that nano-EMPs are greatly outnumbered by non-fibrous nanoparticles across all brain regions.

Starting from deposits in the nose, nano-EMPs appear to translocate—similar to spherical nanoparticles—via the rostral portal of entry to the olfactory bulb and amygdala and via the caudal portal of entry to the trigeminal ganglion (Figure 1). Both pathways have been previously described for the uptake and delivery of drugs *in vitro* and to deeper brain regions in rodent models (Teleanu et al., 2018; Faherty et al., 2025; You et al., 2022), but not for air pollution-derived nano-EMPs. Since we were able to trace the same types of nano-EMPs (e.g., nano-TiO_2_ and nano-amphibole) in different brain regions, including the olfactory bulb, amygdala, and cerebellum (Figure 2), we suggest two distinct “nose-to-brain” pathways are involved in neuronal nano-EMP translocation and subsequent deposition in the olfactory mucosa and respiratory mucosa (Figure 1a). Our study provides the first proof-of-concept that airborne nanosized EMPs, similar to non-elongated nanoparticles (Oberdörster et al., 2005; Aust et al., 2011; Wylie and Korchevskiy, 2023), must be considered separately in studies investigating exposure-dose-response relationships and the potential effects of EMPs on neuroinflammation and neurodegeneration.

The findings from the current study indicate that (a) the OBs in human subjects after lifelong environmental exposure contain nano-EMPs together with a host of non-fibrous nanoparticles, consistent with a functional neuronal pathway for these ambient invader air contaminants from the nose to the OB; (b) further neuronal translocation to the amygdala of the same types of nano-EMPs is observed with HRSTEM (Figure 2); (c) CA bodies collect (internalize) nano-EMPs (Figure 7) and other brain debris; and (d) endogenous biomineralized ferritin iron nanoparticles are co-localized with the nano-EMPs (Figure 5). There are also several mysteries to be solved, including (a) how the larger-sized plastic fibers (>300 nm < 1 μm) enter the cerebellum (Figure 6) and how this compares to the even much larger plastic microfibers (micron-scale; up to 24.4 μm) found by Amato-Lourenco et al. (2024) inside the OB; (b) whether a leaky BBB provides EMPs (including nano- and micro-plastic fibers) access to different regions in the CNS; (c) whether the clearance of EMPs is the same as that of other nanosized particles in the CNS; (d) what mechanisms control the bioprocessing of EMPs; and (e) whether shape controls particle–tissue interactions and reactivity.

In this study, we confirm the olfactory pathway for nano-EMPs and additionally show that nano-EMPs reach the cerebellum of human subjects, which is not only consistent with transport via the trigeminal neuronal translocation pathway (Figure 1a) but also consistent with the entry from the blood circulation (Figure 1b) via leaky capillary junctions across the BBB (Saraiva et al., 2016). Additional translocation routes to the CNS may be discovered for airborne EMPs in the future, e.g., across the cornea by transport via the optic nerve, as hypothesized by Stoddart et al. (2024) and Abbasi et al. (2025) based on their observations in rodents. The latter findings have not been confirmed in humans, and therefore, we did not include the optic nerve transport mechanism in the schematic of the “nose-to-brain” neuronal particle pathways (Figure 1). We also have not yet examined the presence of nano-EMPs in the trigeminal ganglia of the same human subjects shown in Table 1. Nor is there *in vivo* confirmation in humans of EMPs crossing the BBB, although it has been reported that the integrity of the BBB is affected by age and under inflammatory conditions (Knox et al., 2022; Delaney and Campbell, 2017). Physiologically, the BBB provides an important protective function for the brain. All brain tissues are well shielded from even nanoscale particulates by tight endothelial junctions; however, increased leakiness in the ventricle area and in disease states occurs (Saraiva et al., 2016; Knox et al., 2022). Still, whether EMPs can cross the BBB *in vivo* needs to be verified in future studies.

Although we confirmed with correlative microscopy and IHC analyses that the nano-EMPs associate with CA bodies (Figure 7) and accumulate near efflux vessels, including glymphatics and capillaries (Lee et al., 2023), it remains a mystery to what extent nano-EMPs enter CNS tissues in the olfactory or trigeminal pathways or, likely, via a “leaky” BBB at older age and with inflammatory neurogenic diseases (Delaney and Campbell, 2017), or a combination of these. However, although we can be certain that the source of nano-EMPs is of ambient origin when discovered outside the blood compartment in different tissues of the CNS, we cannot provide a definite answer as to their individual transport pathway to their present location nor as to what fraction is due to each of the three translocation pathways shown in Figure 1. Since a compromised BBB is a likely entry portal, especially with age (all 11 subjects were older adults; Table 1), we included the BBB pathway in Figure 1b. Important future work must investigate the ventricular areas and brain endothelial tight junctions in general, along with the trigeminal nerve, for any presence of EMPs at both the nano and microscale.

Another proven fact is that our microscopic evidence shows copious EMPs near neurons with significant myelin damage (Figure 4). There are no direct links to neuronal breakdown from the EMP presence. The Human brain tissues from the 11 subjects (Table 1) contain many other metal and metal oxide nanoparticles (Graham et al., 2020), which creates an overlap regarding the role EMPs play in inducing oxidative stress and neuronal effects. However, our correlative HRSTEM–IHC study confirmed a significant amount of myelin debris with EMPs that were imaged inside CA bodies (Figures 2‐7).

No data exist yet for potential effects of EMPs translocated to the brain that could be used to determine whether the paradigm of “dose, dimension, and durability” that applies to fibers retained in the respiratory tract will also be applicable to EMP effects in brain regions. It has to be considered that the dimensions of EMPs in the CNS are on the nanoscale only, and hence, very different mechanistic aspects have to be carefully taken into account vis-à-vis those in the respiratory tract. Our results are a reflection of the presence of ambient airborne nano-EMPs (metals, carbons, and plastics), which, upon inhalation, translocate to the human OB, amygdala, and cerebellar regions via two portals of entry, one at the beginning of the olfactory and the other at the beginning of the trigeminal pathway. Even nano-plastics are directly emitted in high numbers and mass concentrations into the ambient air by industrial processes (Morales et al., 2022), and an example of nano-plastic fiber presence in the human OB and cerebellum is shown in Figures 5e, 6, respectively. However, as indicated above, it remains uncertain whether the larger-sized EMPs discovered in the cerebellums of our study subjects (e.g., subject #7; Figure 6) arrived via the trigeminal route or by crossing the BBB through the brain capillaries.

Extensive myelin fragmentation (demyelination and phosphorylation) and presence of CAs and ferritin accumulation are commonly observed in all brain tissues that contained a broad range of EMPs, similar to the non-fibrous nanoparticles we reported previously (Graham et al., 2020). Furthermore, we have analytical evidence from HRSTEM/EDS mapping (e.g., Figure 5) of EMP–tissue-related bioprocessing, showing a physicochemical breakdown of the fiber and a partial erosion of the EMP fiber surface with ion shedding into the surrounding brain tissue regions, including those in the OB, amygdala, and cerebellum (Figures 2, 3, 5). Bioprocessing and associated *in vivo* transformations of EMPs cause metal ion imbalances at the EMP–tissue interface together with ferritin nanoparticle buildup (Figures 5e–h). These ferritin clusters (biomineralized iron nanoparticles) further contribute to the already accepted mechanisms by which excess iron accumulates in the brain (Benarroch, 2009). Whether the accumulation of translocated EMPs is a risk factor for neurodegenerative disease requires a more complete understanding of the underlying mechanisms of nano-EMP–tissue interactions. Specifically, the carbon-rich (e.g., proteinaceous/carbonaceous) corona formation around many EMP inclusions (Figure 4) suggests the buildup and selective sequestration of essential carbon-based molecules onto the surfaces of EMPs, thereby affecting vital roles for various functions such as neuronal communication, structural tissue stability, and accessibility of carbonaceous molecules, including proteins, nucleic acids, lipids, and carbohydrates.

## Conclusion

In conclusion, we used advanced analytical imaging techniques coupled with immunohistology methods to map the presence and any biological interactions of nanoscale EMPs originating from ambient air pollution exposure that were found in subcellular compartments within different brain regions. We optimized immunostaining to correlate with neural networks that are nearby nano-EMPs observed in electron microscopy to better understand the biological response. Collectively, we show that the combination of HRSTEM–IHC is a suitable approach for distinguishing nano-EMPs in the cellular environment of the brain. Our results confirmed that airborne ambient nano-EMPs (metals, carbons, and plastics) are present and that these have translocated as inhaled exogenous nanoparticles from the nose to human OB, amygdala, and cerebellar regions via two neuronal pathways, i.e., the olfactory pathway and the trigeminal pathway. The facts that nano-EMPs are found both inside and outside of CAs, within proximity of myelin damage, and that they are surrounded by abundant ferritin nanoparticles are consistent with our hypothesis that nano-EMPs function as invader particles (exogenous) and exert inflammatory responses and that biomineralized ferritin is the body’s response to counter inflammation (Graham et al., 2014; Moreira et al., 2020). However, it remains uncertain whether the EMPs discovered in the cerebellum of our study subjects arrived via the trigeminal route or by crossing the BBB. Particularly, the discovery of the larger EMP fibers (both inorganic and plastic) in the cerebellum questions the likelihood of whether these extraordinarily large EMPs could have been transported via axonal movement in the trigeminal pathway, and we point to the reported probability that inflammatory conditions and age cause extensive capillary BBB leakiness, in particular around ventricular spaces (Knox et al., 2022).

We conclude that although the finding of ambient nano-EMPs in the OB, olfactory tract, amygdala, and cerebellum of human brains is consistent with neuronal translocation from nasal deposits of inhaled nanofibers to the human CNS, in this study, we emphasize the prime importance of verifying the crossing of a “leaky” BBB endothelium by nanoparticles, including nano-EMPs, from the blood compartment and other potential routes to and within the CNS in future studies. The discovery of nano EMPs in different sections of the brain must therefore await a dedicated study using human autopsied brains to investigate EMP translocation across the BBB.

## Data Availability

The original contributions presented in the study are included in the article/supplementary material, further inquiries can be directed to the corresponding author.
